# The Role of Wnt/β-Catenin Signaling and K-Cadherin in the Regulation of Intraocular Pressure

**DOI:** 10.1167/iovs.17-21964

**Published:** 2018-03

**Authors:** Hannah C. Webber, Jaclyn Y. Bermudez, J. Cameron Millar, Weiming Mao, Abbot F. Clark

**Affiliations:** North Texas Eye Research Institute, University of North Texas Health Science Center

**Keywords:** Wnt signaling, cadherins, intraocular pressure, glaucoma, trabecular meshwork, mouse

## Abstract

**Purpose:**

Wnt/β-catenin signaling in the trabecular meshwork (TM) is required for maintaining normal intraocular pressure (IOP), although the mechanism(s) behind this are unknown. We hypothesize that Wnt/β-catenin signaling regulates IOP via β-catenin's effects on cadherin junctions.

**Methods:**

Nonglaucomatous primary human TM (NTM) cells were treated with or without 100 ng/ml Wnt3a, 1 μg/ml sFRP1, or both for 4 to 48 hours. Cells were immunostained for β-catenin, total cadherins, or cadherin isoforms. Membrane proteins or whole-cell lysates were isolated for Western immunoblotting and probed for cadherin isoforms. RNA was extracted for cDNA synthesis and qPCR analysis of cadherin expression. Some NTM cells were cultured on electric plates for cell impedance assays. Ad5.CMV recombinant adenoviruses encoding K-cadherin, and/or sFRP1 were injected into eyes of 4- to 6-month-old female BALB/cJ mice (*n* = 8–10). Conscious IOPs were assessed for 35 days.

**Results:**

Upon Wnt3a treatment, total cadherin expression increased and β-catenin accumulated at the TM cell membrane and on processes formed between TM cells. qPCR showed that Wnt3a significantly increased K-cadherin expression in NTM cells (*P* < 0.01, *n* = 3), and Western immunoblotting showed that Wnt3a increased K-cadherin in NTM cells, which was inhibited by the addition of sFRP1. Cell impedance assays showed that Wnt3a treatment increased transcellular resistance and anti-K-cadherin siRNA decreased transcellular resistance (*P* < 0.001, *n* = 4–6). Our in vivo study showed that K-cadherin significantly decreased sFRP1-induced ocular hypertension (*P* < 0.05, *n* = 6). Western immunoblotting also showed that K-cadherin alleviated sFRP1-induced β-catenin decrease in mouse anterior segments.

**Conclusions:**

Our results suggest that cadherins play important roles in the regulation of TM homeostasis and IOP via the Wnt/β-catenin pathway.

Glaucoma is a blinding disease characterized by optic nerve degeneration. Glaucoma is age-associated, and a study from the 2005–2008 National Health and Nutrition Examination Survey (NHANES) cohort shows that 2.91 million Americans over the age of 40 years suffer this disease.^1^ As the average life span continues to rise, the prevalence of glaucoma is expected to increase. Primary open angle glaucoma (POAG) is the most common form of glaucoma, and a meta-analysis of 81 studies on POAG prevalence showed that in 2015, 57.5 million people globally were affected by POAG.^2^ This number is expected to rise to 65.5 million by 2020,^2^ with some 11.1 million people being bilaterally blind (150,000 in the United States alone).^1,2^ POAG is a heterogeneous disease associated with multiple risk factors including ethnicity, family history, aging, and ocular hypertension (OHT).^3^ OHT is defined by an intraocular pressure (IOP) of 21 mm Hg or higher and is the main causative and currently the only clinically modifiable risk factor. In POAG patients with OHT, IOP increases when aqueous humor outflow resistance at the trabecular meshwork (TM) increases, and increased outflow resistance is due to pathological changes in the TM. However, there are no currently available therapeutics that directly target TM pathology, and the causes of these pathological changes to the TM are still under investigation. When compared with nonglaucomatous TM (NTM), glaucomatous TM tissues show decreased TM cellularity, increased extracellular matrix (ECM) in trabecular beams and juxtacanalicular tissue, increased TM stiffness, increased actin contractility, increased cross-linked actin networks, and dysregulation of TM cell signaling pathways including the transforming growth factor beta (TGFβ), bone morphogenetic protein, and Wnt/β-catenin signaling pathways.^4–7^ Many studies showed that increased expression of the growth factors including TGFβ2, gremlin, and secreted frizzled-related protein 1 (sFRP1) can cause dysregulation of these homeostatic pathways in the glaucomatous TM.^8–15^

sFRP1 is an antagonist of the Wnt signaling pathway that sequesters extracellular Wnt ligands, thereby blocking their binding to frizzled receptors.^16,17^ In the absence of Wnt ligands, the intracellular “Wnt/β-catenin inhibitory complex” made up of glycogen synthase kinase 3 beta (GSK-3β), Axin, APC, and casein kinase 1 (CK1) proteins tag the Wnt/β-catenin signaling mediator β-catenin for destruction through phosphorylation, ubiquitination, and proteasomal degradation. Therefore, β-catenin will be constantly degraded in the cell and will cease to translocate into the nucleus and will be unable to regulate transcription via binding at the T-cell factor/lymphoid enhancer factor promoter.^18^ Our previous studies showed that the expression of sFRP1 is increased in glaucomatous TM cells.^10^ We have shown that a number of Wnt pathway genes are expressed in the human TM (HTM), and the Wnt ligand Wnt3a activates the Wnt/β-catenin signaling pathway.^10,19^ Overexpression of sFRP1 or Dkk1 (a specific Wnt/β-catenin signaling inhibitor) leads to increased IOP in perfusion-cultured human eyes and in mouse eyes.^10,19^ We also found that cotreatment with a small-molecule Wnt pathway activator inhibits sFRP1-induced OHT in mouse eyes. Indirect activation of Wnt/β-catenin signaling in the TM using lithium chloride (LiCl) was reported by the Rhee group to decrease the production of some ECM and matricellular proteins as well as inhibit the TGFβ pathway and TGFβ2-induced ECM production.^20^ Sun et al.^21^ showed that intraperitoneal administration of LiCl decreased IOP in a rat OHT model. However, this has been the only report showing the ocular hypotensive effect of LiCl and further studies are needed to determine the mechanism of LiCl; that is, whether LiCl functions via the Wnt/β-catenin signaling pathway and/or other signaling pathways.^21^ We have shown that the Wnt/β-catenin and TGFβ/Smad pathways cross-inhibit each other in NTM cells, and this cross-inhibition relies on the key pathway transcription factors β-catenin and Smad4.^22^ However, the mechanism by which Wnt/β-catenin signaling regulates IOP remains unclear.

β-catenin is the key transcription factor for Wnt/β-catenin signaling, but it has another function in the cell as an accessory protein for classical cadherin junctions. Cadherins are transmembrane proteins whose extracellular domains bind homophilically to their counterparts on other cells in a calcium-dependent manner.^23^ The classical cytosolic domain of cadherins binds directly to β-catenin, among other accessory proteins such as α-catenin and p120-catenin.^24^ Cadherins are connected to and stabilized by the actin cytoskeleton through these catenin accessory proteins, and β-catenin is essential for the cadherin-actin complex. Phosphorylation of β-catenin at different sites and the localization of β-catenin determine whether β-catenin remains bound to cadherins, and the lack of β-catenin-cadherin binding disrupts the cadherin-β-catenin-actin complex.^25,26^ Because β-catenin plays major roles in both Wnt/β-catenin signaling and adherens junctions, it is likely that Wnt/β-catenin signaling regulates the anchoring of cadherins junctions to the actin cytoskeleton by regulating membrane associated β-catenin. Currently, the connection between Wnt/β-catenin signaling and cadherins junctions has not been explored in the TM.

Cadherins play key roles in cell-cell adhesion, directing tissue patterning and forming neural connections during development, epithelial to mesenchymal transition in cancers and wound healing, thereby serving as mechanosensors that relay mechanical signals from the cells' environment to the actin cytoskeleton to facilitate a tensile or migratory response.^27–30^ Many isoforms of cadherin proteins exist in multiple species including humans, and their expression and distribution are highly tissue specific. Different cadherin isoforms convey different physiological properties to cells. Wecker et al.^31^ showed that in human TM cells, N-cadherin and OB-cadherin are up-regulated by TGFβ2. However, the expression of the other cadherin isoforms and the effect of Wnt signaling on cadherins in the TM are still unclear. In addition, the role of cadherins in IOP regulation is unknown.

In this study, we determined the expression and localization of a subset of cadherins in primary NTM cells and the regulation of these cadherins by Wnt/β-catenin signaling. We also studied the role of cadherins and Wnt signaling on cell adhesion using real-time cell analysis (RTCA) impedance assays, as well as the role of cadherins in sFRP1-induced OHT in mice. We hypothesize that Wnt/β-catenin signaling activation increases the expression of cadherins in TM cells, contributing to TM cell adhesion, and that the inhibition of Wnt/β-catenin signaling compromises cadherin expression as well as TM cell adhesion and mechanotransduction, thereby inducing OHT.

## Methods

### NTM Cells

Primary NTM cells were characterized and donated by Alcon as described previously.^32,33^ The cell strains used in these experiments were derived from human donor eyes and included: NTM895-03, female, age 63 years; NTM176-04, male, age 72 years; NTM875-03, male, age 77 years; NTM496-05, female, age 82 years; NTM1022-02, male, age 67 years; NTM444-05, male, age 85 years.

### Immunocytofluorescence (ICF)

NTM cells were cultured on glass coverslips in DMEM-low glucose medium supplemented with 10% fetal bovine serum, 1% glutamine, and 1% penicillin and streptomycin (Thermo Fisher Scientific, Waltham, MA, USA). When cells were confluent, culture medium was changed to serum-free medium, and the cells were cultured for an additional 24 hours. The cells were then treated with or without 100 ng/ml recombinant Wnt3a and/or 1 μg/ml recombinant sFRP1 (R&D Systems, Minneapolis, MN, USA) for 24 hours. At the end of treatment, cells were fixed with 4% paraformaldehyde at 4 °C, treated with 0.05% Triton X-100, blocked with Superblock (Thermo Fisher Scientific) and immunostained with rabbit anti-β-catenin (1:250; Cell Signaling Technology, Danvers, MA, USA), rabbit anti-K-cadherin (1:250; Abcam, Cambridge, UK) or rabbit anti-Pan-cadherin antibodies (1:250; Abcam). After incubation with a secondary donkey anti-rabbit antibody conjugated with Alexafluor 488 (1:500; Thermo Fisher Scientific), some cells were stained with with Phalloidin conjugated with Alexafluor 594 (1:1000; Life Technologies, Carlsbad, CA, USA) for 1 hour. After staining, glass coverslips were mounted on slides using the ProLong gold antifade reagent with 4′,6-diamidino-2-phenylindole (DAPI; Life Technologies). All images were captured using a Nikon Eclipse Ti-U Epifluorescent microscope (Nikon, Minato, Tokyo, Japan) in combination with a Nuance imaging system (Nuance Communications, Burlington, MA, USA).

### Quantitative Polymerase Chain Reaction (qPCR)

NTM cells were cultured in 12-well plastic dishes until they were confluent. They were serum-starved for 24 hours, then treated for an additional 24 hours with or without 100 ng/ml Wnt3a and/or 1 μg/ml sFRP1. Triplicates were used for each treatment. After treatment, total RNA was isolated using the Qiagen RNeasy Mini Kit (Qiagen, Hilden, Germany). RNA was reverse transcribed into cDNA using iScript Reverse Transcription Supermix (Bio-Rad, Hercules, CA, USA), and qPCR was performed using the SsoAdvance Universal SYBR Green Supermix (Bio-Rad) in a CFX96 thermocycler (Bio-Rad). The thermoprofile consisting of 40 cycles of 95°C for 10 seconds, 60°C for 30 seconds, followed by a temperature dissociation curve. GAPDH primers were used as an internal control. All the primers used were designed to span or flank exon-exon junctions. The primer sequences (synthesized by Sigma- Aldrich, St. Louis, MO, USA) are as follows:

- Axin2:

Forward: CAGATCCGAGAGGATGAAGAGA; reverse: AGTATCGTCTGCGGGTCTTC

- K-cadherin:

Forward: GGCAGATCAGTTGATTCAGA; reverse: GCCGTGTTGTCTTTGTTGTC

- OB-cadherin:

Forward: CAAAGTTTCCGCAGAGCGTA; reverse: GCTTTATCACCCCCTCCTGT

- N-cadherin:

Forward: ATCCTGCTTATCCTTGTGCTG; reverse: CCTGGTCTTCTTCTCCTCCA

- GAPDH^34^:

Forward: GGTGAAGGTCGGAGTCAAC; reverse: CCATGGGTGGAATCATATTG

- Cadherin-19 A:

Forward: TGCAGGCTCTGGTCAGGTA; reverse: GAAGACAGGTTCTTCTTGAAGGTT

- Cadherin-19 B:

Forward: AGCACAAGCGTCTGTAACTCTG; reverse: GGAAACAGGACGTCACTAACAA.

### Western Immunoblotting (WB)

NTM cells were cultured in 60-mm dishes until they were confluent. The cells were serum-starved for 24 hours and treated with 100 ng/ml Wnt3a and/or 1 μg/ml sFRP1 for an additional 4, 24, or 48 hours. After treatment, the membrane protein fraction was isolated using the Eukaryotic Membrane Extraction Kit (Thermo Fisher Scientific), while total protein was isolated using the M-PER Mammalian Extraction Reagent (Thermo Fisher Scientific). Equal amounts of protein from each sample were separated using SDS-PAGE, transferred onto an Immobilon-P transfer membranes, blocked with 5% dry milk, and immunoblotted overnight at 4°C with rabbit anti-K-cadherin antibody (1:500; Abcam), rabbit anti-OB-cadherin antibody (1:500; Abcam), rabbit anti-β-catenin antibody (1:500; Cell Signaling), rabbit anti-GAPDH antibody (1:10,000; Cell Signaling), or mouse anti-Lamin A/C antibody (1:1000; Cell Signaling). The blots were then washed and incubated in secondary horse radish peroxidase (HRP)-linked anti-rabbit or anti-mouse IgG antibodies (1:5000; Cell Signaling Technology). Signals were developed using the Clarity Western ECL Blotting substrate (Bio-Rad). Images were taken using the Bio-Rad ChemiDoc imaging system (Bio-Rad). For some blots, densitometry was performed using ImageJ (National Institutes of Health, Bethesda, MA, USA).

### Transfection

NTM cells were grown to 80% confluency in 8- or 12-well Acea electric plates containing copper electrodes. The transfection mix contained 1nM anti-K-cadherin, anti-OB-cadherin, or nontargeting siRNAs (OnTarget siRNA; GE Dharmacon, Lafayette, CO, USA) along with 0.83 μl Attractene (Qiagen) per well for Acea plates and 5 μl Attractene per well for regular 12-well plates. Attractene and siRNA were incubated together in a transfection mix in OptiMEM (Thermo Fisher Scientific) for 20 minutes. Trypsinized NTM cells were resuspended in OptiMEM containing 5% FBS and 1% glutamine at a density of 2 × 105/ml and 200 uL of transfection mix was added. Eighteen to 24 hours after transfection, culture medium was changed to Dulbecco's modified Eagle medium (DMEM) low glucose containing 10% FBS, 1% glutamine, and 1% penicillin/streptomycin. NTM cells in 12-well plates were harvested 72 hours after treatment for WB. NTM cells cultured in 8-well Acea plates were placed on the Acea iCelligence system for continuous cell impedance measurements.

### RTCA Cell Impedance Assay

The RTCA iCELLigence system (Acea Biosciences, San Diego, CA, USA) was used for NTM cell impedance (electric resistance) measurements. This system has been described previously.^35^ Briefly, this assay measures impedance in real time of live cells cultured in specialized copper electrode-coated plates. The cell index (CI) is used to represent the maximum change in cell impedance during a specific time frame.

\newcommand{\bialpha}{\boldsymbol{\alpha}}\newcommand{\bibeta}{\boldsymbol{\beta}}\newcommand{\bigamma}{\boldsymbol{\gamma}}\newcommand{\bidelta}{\boldsymbol{\delta}}\newcommand{\bivarepsilon}{\boldsymbol{\varepsilon}}\newcommand{\bizeta}{\boldsymbol{\zeta}}\newcommand{\bieta}{\boldsymbol{\eta}}\newcommand{\bitheta}{\boldsymbol{\theta}}\newcommand{\biiota}{\boldsymbol{\iota}}\newcommand{\bikappa}{\boldsymbol{\kappa}}\newcommand{\bilambda}{\boldsymbol{\lambda}}\newcommand{\bimu}{\boldsymbol{\mu}}\newcommand{\binu}{\boldsymbol{\nu}}\newcommand{\bixi}{\boldsymbol{\xi}}\newcommand{\biomicron}{\boldsymbol{\micron}}\newcommand{\bipi}{\boldsymbol{\pi}}\newcommand{\birho}{\boldsymbol{\rho}}\newcommand{\bisigma}{\boldsymbol{\sigma}}\newcommand{\bitau}{\boldsymbol{\tau}}\newcommand{\biupsilon}{\boldsymbol{\upsilon}}\newcommand{\biphi}{\boldsymbol{\phi}}\newcommand{\bichi}{\boldsymbol{\chi}}\newcommand{\bipsi}{\boldsymbol{\psi}}\newcommand{\biomega}{\boldsymbol{\omega}}\[{\rm{CI}}=\mathop {\max }\limits_{i = 1, \ldots ,N} \left[ {{{{R_{{\rm{cell}}}}\left( {f_i} \right)} \over {{R_{\rm{b}}}\left( {f_i} \right)}} - 1} \right]{}\]

*R*_cell_(*f_i_*) is the measured electric resistance/impedance at a certain time point in wells with cells.

*R*_b_(*f_i_*) is the measured electric resistance/impedance at a certain time point in wells without cells.

CI was calculated every hour. “Normalized CI” represented CI values compared to a CI value chosen by the researcher and that CI was set at 1. In these experiments, we chose to normalize the CI to a time point within 2 hours before treatment, which was indicated by the black bar in the iCelligence software screenshot (Supplementary Fig. S2).

NTM cells were seeded on Acea copper electrode-coated plates in replicates (*n* = 4–6). Baseline CI values were collected every hour for at least 48 hours. NTM cells were then either treated with recombinant proteins or transfected with siRNA, and CI values were collected every hour for 72 to 96 additional hours. Recombinant protein treatment regime included control, 100 ng/ml Wnt3a, 1 μg/ml sFRP1, or both. The averaged maximum and minimum CI values for each treatment group during this 72-hour time period were presented. For transfection experiments, NTM cells were transfected with anti-K-cadherin siRNA (*n* = 6), anti-OB-cadherin siRNA (*n* = 6), or nontargeting siRNA (*n* = 4), and CI values were collected every hour for 96 hours. The averaged maximum and minimum CI values during the last 72 hours of this time period were presented because siRNA treatment typically takes 24 hours to knockdown expression of the targeted mRNA.

### Adenoviral Vectors

Adenovirus serotype 5 (Ad5) vectors that overexpress the human K-cadherin and mCherry (Ad5.K-cadherin), mouse sFRP1 (Ad5.sFRP1), as well as a null vector (Ad5.Null) were obtained commercially from Vector Labs (Burlingame, CA, USA). The expression of each exogenous gene was driven by its own cytomegalovirus (CMV) promoter, including mCherry in the Ad5.K-cadherin vector.

### Viral Transduction

All mouse studies were conducted in compliance with the University of North Texas Health Science Center Institutional Animal Care and Use Committee and the ARVO Statement on the Use of Animals in Ophthalmic and Vision Research. Female BALB/cJ mice were purchased from The Jackson Laboratory (Bar Harbor, ME, USA). Mice aged 4 to 6 months were used for intravitreal adenoviral injection. Prior to use, all animals' eyes were examined using a hand-held ophthalmoscope (Welch-Allyn, Skaneateles Falls, NY, USA) to confirm a normal appearance. Immediately before viral injection, mice were anesthetized with a cocktail of ketamine/xylazine (100 mg/kg and 10 mg/kg, respectively) administered intraperitoneally. Some of the mice were instead anesthetized using inhalation anesthesia (isoflurane [2.0%–2.5%], in combination with O_2_ [0.8 L/min]). After anesthesia, equal numbers of infectious units (IFU) were injected into the vitreous chamber of left eyes, 3 × 10^7^ infectious units in 1 to 5 μl were slowly injected during a period of 1 to 2 minutes using a glass microsyringe (Hamilton Company, Reno, NV, USA) fitted with a 33-G needle. The uninjected right eyes served as paired controls.

The treatment groups were as follows with each group composed of 8 to 10 mice: Ad5.K-cadherin (1.5 × 10^7^ IFU) + Ad5.sFRP1 (1.5 × 10^7^ IFU); Ad5.K-cadherin (1.5 × 10^7^ IFU) + Ad5.Null (1.5 × 10^7^ IFU); Ad5.sFRP1 (1.5 × 10^7^ IFU) + Ad5.Null (1.5 × 10^7^ IFU); and Ad5.Null (3 × 10^7^ IFU).

To determine if viral transduction of K-cadherin interfered with sFRP1 expression, NTM cells were transduced with Ad5.Null, Ad5.sFRP1, Ad5.K-cadherin, or Ad5.sFRP1+Ad5.K-cadherin at the multiplicity of infection of 100. Transduced cells were harvested 3 days after transduction for WB analysis.

### Conscious Mouse IOP Measurement

Conscious mouse IOP was assessed in a masked manner using a TonoLab rebound tonometer (Colonial Medical Supply, Franconia, NH, USA). Baseline IOP was measured before viral injection, and IOP post–viral injection was monitored on indicated days. At days 9 and 21, two mice from the Ad5.K-cadherin, Ad5.sFRP1, and Ad5.K-cadherin + Ad5.sFRP1 groups were sacrificed for subsequent analyses.

### Mouse Tissue Analyses

The mice were euthanized using carbon dioxide. Immediately afterward, the mouse eyes were enucleated and placed in ice-cold PBS.

The anterior segments of some eyes were dissected using a dissection microscope by first cutting around the equator of the eye and removing the posterior segment. The lens, ciliary body, and iris were then removed from the anterior segment, leaving only the cornea and the tissues of the conventional outflow pathway. This tissue, which we refer to as the anterior segment, was placed in the T-PER Tissue Protein Extraction Reagent (Thermo Fisher Scientific) with 1:100 protease inhibitors. Protein was extracted using the Tissuelyser (Qiagen). An equal amount of protein from each sample was separated using SDS-PAGE, transferred onto an Immobilon-P transfer membranes, blocked with 5% dry milk, and immunoblotted with anti-mCherry (1:500, Abcam), anti-β-catenin (1:500, Cell Signaling Technology), or anti-GAPDH (1:2000, Cell Signaling Technology) antibodies, overnight at 4°C. The membranes were rinsed and then incubated in secondary HRP-linked antirabbit or antimouse IgG antibody (1:5000, Cell Signaling Technology). Signals were developed using the Clarity Western ECL Blotting substrate (Bio-Rad). Images were taken using the Bio-Rad ChemiDoc imaging system (Bio-Rad).

The other eyes were fixed with 4% paraformaldehyde at 4°C for 2 hours. Fixed eyes were washed with PBS, dehydrated, embedded in paraffin, sectioned, and mounted on glass slides. Some sections were subjected to hematoxylin and eosin staining. The other sections were immunostained for sFRP1, mCherry, and β-catenin. These tissue sections were baked at 60°C for 30 minutes, dewaxed, and rehydrated through a series of incubations with Xylene, 100% ethanol, 95% ethanol, 50% ethanol, and water. Tris-EDTA buffer (pH 9.0) was used for antigen retrieval together with the 2100 Retriever (Electron Microscopy Sciences, Hatfield, PA, USA). After antigen retrieval, the sections were treated with 0.5% Triton X-100, blocked with Superblock Blocking Buffer (Thermo Fisher Scientific), and incubated with 1:100 rabbit anti-sFRP1 antibody (Novus Biological, Littleton, CO, USA) overnight at 4°C. The sections were then incubated with the donkey antirabbit secondary antibody conjugated with Alexafluor 488 (Thermo Fisher Scientific, 1:500) at room temperature for 2 hours, and mounted using the ProLong gold antifade reagent with DAPI (Life Technologies). Images were taken using the Nikon Eclipse Ti-U Epifluorescent microscope (Nikon, Melville, NY, USA) and the Nuance imaging system (PerkinElmer, Hopkinton, MA, USA).

### Statistics

One-way ANOVA was used for analysis, and *P* values less than 0.05 were considered significant.

## Results

### The Wnt Signaling Pathway Regulated Membrane-Associated β-Catenin and Total Cadherins in TM Cells

**Figure 1:**
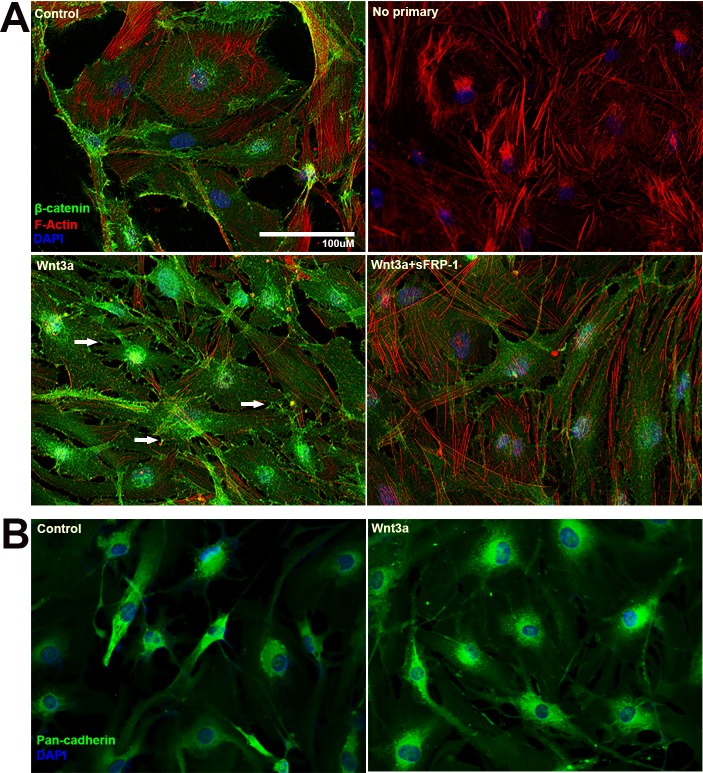
Wnt signaling activation increased membrane-associated β-catenin and total cadherin in primary NTM cells. Primary NTM cells were treated with or without Wnt3a and/or sFRP1 for 24 hours in serum-free medium before fixation and ICF. (A) NTM cells were stained for β-catenin (green), F-actin (red), and nuclei (blue) using antibody, phalloidin-Alexa 594 and DAPI, respectively. White arrows indicate filopodia-like cell-cell connections enriched with membrane-associated β-catenin. (B) ICF staining of total cadherins (green) and nuclei (blue).

We first determined whether the expression of β-catenin and cadherin is regulated by Wnt signaling. NTM cells were treated with or without 100 ng/ml Wnt3a and/or 1 μg/ml sFRP1 for 24 hours^19^ and harvested for ICF to study the distribution of β-catenin (Fig. 1A). We found that Wnt3a induced accumulation of cytosolic β-catenin, translocation of nuclear β-catenin, as well as membrane associated β-catenin. Some membrane associated β-catenin was localized to filopodia-like connections between TM cells (Fig. 1A, white arrows).

Because β-catenin is part of the cadherin junctional complex, we then determined whether the Wnt pathway also regulates cadherins in the TM. NTM cells were treated with or without Wnt3a for 24 hours and immunostained using an antibody (anti-Pan-cadherin antibody) that recognizes all isoforms of cadherins (total cadherins; Fig. 1B). We found that Wnt3a slightly increased total cadherin expression and led to a broad distribution of cadherins in the cytoplasm as well as on cell membranes, which suggested that the activation of canonical Wnt signaling affects cadherins and cell adhesion. The high-resolution image in Figure 1B shows the presence of cadherins at cell-cell junctions using an anti-pan-cadherin antibody. TM cells are different from endothelial or epithelial cells, in which there is extensive cell-cell contact and high levels of cadherins. In contrast, TM cells have long overlapping processes and cadherins may be enriched in the processes that form cell-cell junctions with other TM cells. In in vitro cultures, TM cells are two-dimensional and made less contact, which leads to lower apparent cadherin staining.

### The Wnt Signaling Pathway Regulated the Expression of K, OB, and N-Cadherins in the TM

**Figure 2:**
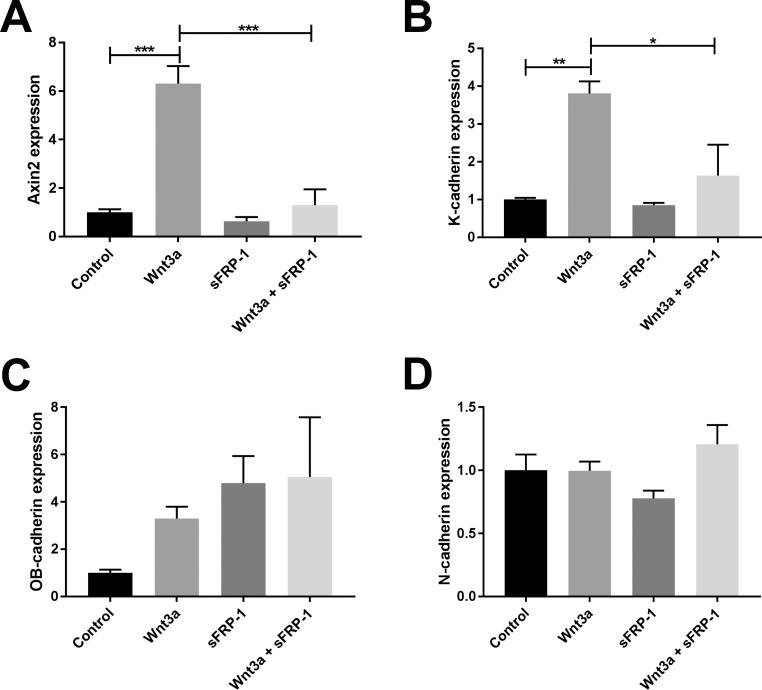
Wnt/β-catenin signaling activation induced K-cadherin mRNA expression. Primary NTM cells were treated with or without Wnt3a and/or sFRP1 for 24 hours, and RNA was extracted for reverse transcription and qPCR. Axin2 was used as a marker of the activation of Wnt/β-catenin signaling. GAPDH was used as an internal control for normalization. Columns and error bars: means and standard error of mean (SEM). One-way ANOVA with the Dunnett post hoc test was used for statistical analysis. N = 3. *P < 0.05, **P < 0.01, ***P < 0.001.

Since the cadherin family contains many genes we determined which cadherins are expressed in the TM. We searched the Iowa Ocular Tissue Database, and found that K-cadherin, OB-cadherin, cadherin19, and N-cadherin are the most abundantly expressed cadherins in HTM tissues.^36^ To confirm the expression of these cadherins in the TM, we performed PCR using RNA isolated from NTM cells. K, OB, and N-cadherins were detected in the TM (Fig. 2). In contrast, cadherin19 was not detected using two different set of primers (data not shown).

We then determined whether the expression of the three cadherins is regulated by the Wnt signaling pathway. We treated NTM cells with or without Wnt3a and/or sFRP1 for 24 hours and compared the levels of K, OB, and N-cadherins using qPCR. Axin2, whose expression is induced by the Wnt pathway, is frequently used as a marker for Wnt signaling activation.^37^ We found that K- and OB-cadherins were significantly increased by Wnt3a treatment (Fig. 2). Cotreatment with sFRP1 completely inhibited Wnt3a-induced K-cadherin expression, but not OB-cadherin. In contrast, Wnt3a did not change the expression of N-cadherin in TM cells. Interestingly, K-cadherin does not seem to have the T-cell factor/lymphoid enhancer factor binding element sequence within its promoter (http://www.sabiosciences.com/chipqpcrsearch.php?app=TFBS, provided in the public domain), so the increase of K-cadherin production may be a secondary effect of Wnt/β-catenin signaling activation.

### Wnt3a Increased Total and Membrane-Associated K-Cadherin Expression in TM Cells

**Figure 3:**
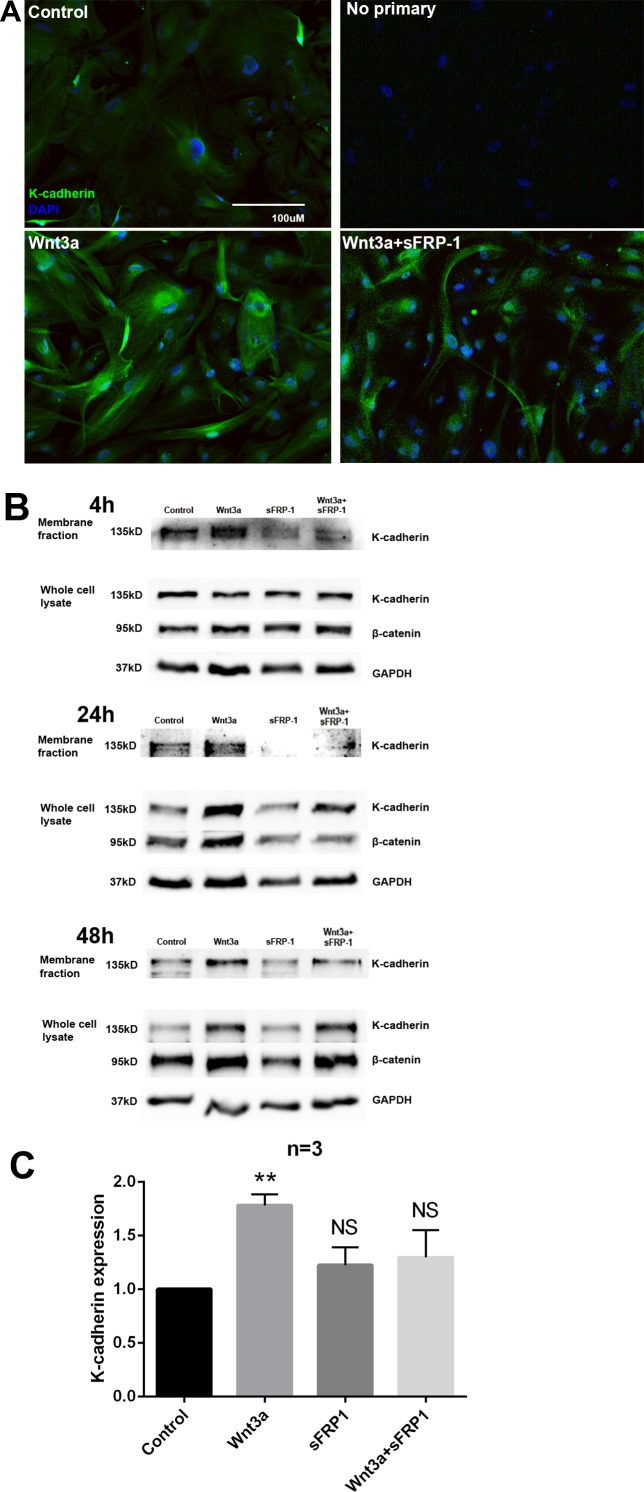
K-cadherin protein expression was regulated by Wnt/β-catenin signaling. (A) Primary NTM cells were treated for 24 hours with or without Wnt3a and/or sFRP1. Cells were fixed and immunostained for K-cadherin (green). Nuclei (blue) were stained using DAPI. (B) Primary NTM cells were treated for 4, 24, or 48 hours with or without Wnt3a and/or sFRP1. Membrane protein fractions or whole-cell lysates were isolated and WBs were probed for K-cadherin, β-catenin, and GAPDH. Representative images are shown. (C) Densitometry analysis of K-cadherin from whole-cell lysate extracted from three NTM cell strains (N = 3) treated with indicated recombinant proteins for 24 hours. **P < 0.01 when compared with control using one-way ANOVA and Dunn's post hoc test.

Because the induction of K-cadherin by Wnt3a correlated with Wnt signaling activation, we further studied whether the distribution of membrane-associated K-cadherin is regulated by the Wnt/β-catenin signaling in the TM. We treated NTM cells with or without Wnt3a and sFRP1 and compared K-cadherin expression using ICF (Fig. 3A). We found that Wnt3a treatment increased the expression of K-cadherin in TM cells. This increased expression of K-cadherin was partially inhibited by cotreatment with sFRP1 (Fig. 3A). We also found a difference in cell morphology including cell size and length between Wnt3a-treated and Wnt3a+sFRP-1-treated NTM cells. Wnt3a-treated NTM cells were normally sized and had long cell processes commonly seen in TM cell cultures. Upon the addition of sFRP-1, TM cells shrank and their processes retracted, possibly due to the loss of K-cadherin-mediated cell adhesion.

To quantitatively compare membrane-associated K-cadherin, we extracted the membrane protein fraction or whole-cell lysate from treated cells for WB analysis (Fig. 3B). Membrane-associated K-cadherin increased 4 hours after Wnt3a treatment, but total K-cadherin did not, which suggested that Wnt signaling alters K-cadherin distribution at an early stage before transcriptional regulation occurs. By 24 hours, total K-cadherin increased significantly with Wnt3a treatment (Figs. 3B, 3C) as well as a more profound accumulation of β-catenin. This increase in membrane-associated and total K-cadherin as well as β-catenin was maintained for 48 hours. All of these inductions were inhibited by cotreatment with sFRP1. These data show that Wnt/β-catenin signaling plays a key role in regulating K-cadherin expression and localization in different intracellular compartments.

### Wnt Signaling and K-Cadherin Regulated TM Cell Impedance

To determine the contribution of K-cadherin to TM cell adhesion, we performed RTCA cell impedance assays. Cell impedance is a measurement of electric resistance across cells that is affected by cell adhesion. As described in the Methods section, CI was calculated to quantify changes in impedance.

**Figure 4:**
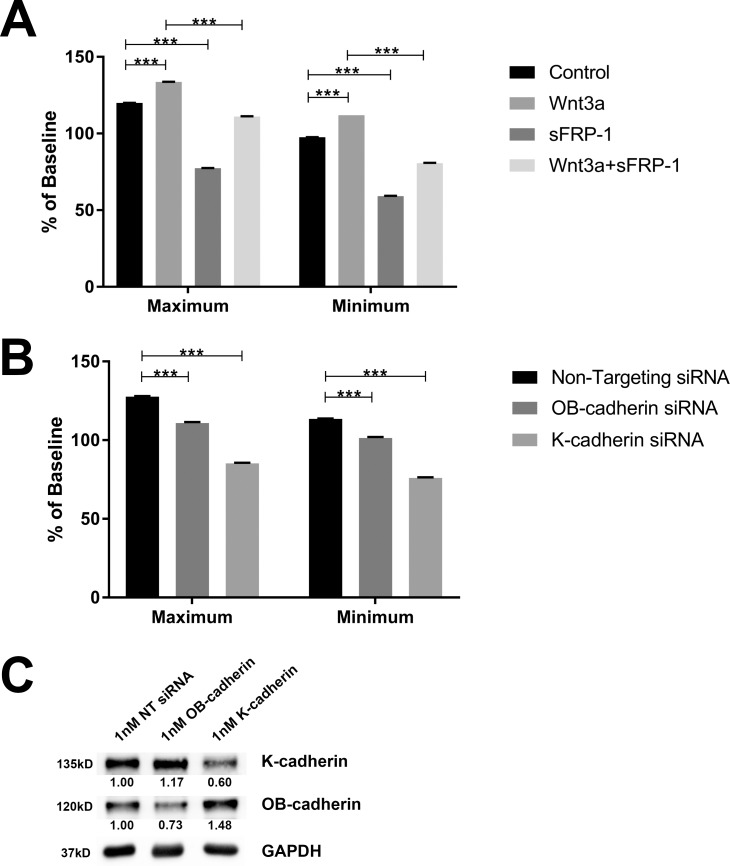
Wnt signaling and K-cadherin regulated HTM cell impedence. Maximum and minimum normalized CI values of NTM cells were collected over 72 hours using the Acea RTCA iCelligence system. (A) NTM cells were treated with or without Wnt3a and/or sFRP1. (B) NTM cells were transfected with 1nM nontargeting (NT), anti-OB-cadherin, or anti-K-cadherin siRNA. (C) siRNA transfected NTM cells were used for WB to determine protein expression levels.

First, we studied how changes in Wnt signaling affects cell impedance. NTM cells were cultured, and cell impedance baselines were established. The cells were then treated with or without Wnt3a and/or sFRP1 for 3 to 4 days. We found that Wnt3a significantly increased both maximum and minimum CI values compared to untreated controls, while the Wnt signaling antagonist sFRP1 significantly decreased CI (both *P* < 0.001, *n* = 4; Fig. 4A). Also, cotreatment with sFRP1 inhibited Wnt3a-induced CI elevation (*P* < 0.001, *n* = 4; Fig. 4A).

Second, we studied the effects of cadherins on NTM cell impedance (Fig. 4B) because we have shown that Wnt signaling affects cadherins, and cadherins are known to be the key players of cell-cell adhesion. We knocked down OB- or K-cadherins in NTM cells using siRNA and achieved a 30% to 40% decrease of the two cadherins proteins, respectively (Fig. 4C). Maximum and minimum CI values were decreased significantly after OB- or K-cadherin knockdown compared to nontargeting siRNA controls (*P* < 0.001, *n* = 4–6; Fig. 4B).

Our findings suggest that Wnt signaling is positively correlated with cell adhesion, which is very likely through the regulation of cadherins.

### K-Cadherin Inhibited sFRP1-Induced OHT

**Figure 5:**
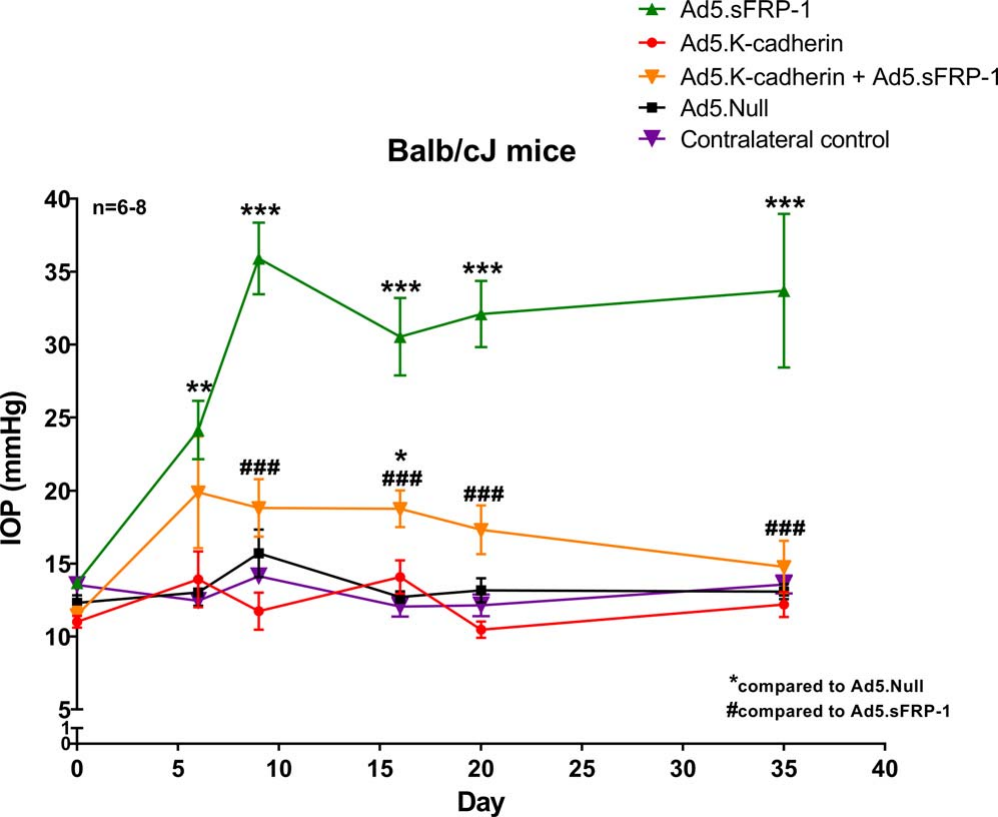
K-cadherin inhibited sFRP1-induced OHT in mouse eyes. BALB/cJ mice were intravitreally injected with 3 × 10^7^ IFU Ad5 viral vectors encoding no foreign genes (Null), human sFRP1, murine K-cadherin, or a mix of two viral vectors (n = 6–7 per group). IOP was measured before injection (day 0, baseline) as well as on days 6, 9, 16, 20, and 35 postinjection. The uninjected eyes were used as the contralateral control (naïve control). One-way ANOVA was performed for each time point with a Tukey post hoc test. *P < 0.05, **P < 0.01, and ***P < 0.001 as compared to Ad5.Null transduced eyes. ###P < 0.001 as compared to Ad5.sFRP1 transduced eyes.

Our published studies showed that the inhibition of Wnt/β-catenin signaling leads to OHT.^10,19^ Because we have shown that Wnt/β-catenin signaling regulates cadherins, we determined whether K-cadherin plays a role in maintaining IOP. We transduced mouse TM (MTM) tissues by injecting Ad5.Null, Ad5.sFRP1, Ad5.K-cadherin, or Ad5.sFRP1+Ad5.K-cadherin virus(es) into the vitreous chamber of the left eyes of 4- to 6-month-old BALB/cJ mice. Viral particle numbers were identical between groups, with some groups also receiving Ad5.Null virus to compensate. The right eye served as an uninjected naïve control. Our published studies have shown that Ad5 has a tropism for the TM.^38^ Conscious IOPs of both eyes were recorded using a rebound tonometer. Baseline IOP at day 0 and postinjection IOPs at indicated days were plotted over time (Fig. 5).

We found that Ad5.sFRP1 induced OHT (*P* < 0.01 or 0.001 compared to Ad5.Null or contralateral control, respectively), which matched our previously published studies.^10,19^ We also found that Ad5.K-cadherin alone did not affect IOP (*P* > 0.05 compared to both controls). However, cotransduction with Ad5.K-cadherin partially but significantly inhibited Ad5.sFRP1-induced OHT from days 6 to 35 (*P* < 0.001 compared to sFRP1 alone), and it completely inhibited OHT at day 35 (*P* > 0.05 compared to controls).

During and at the end of the study, we collected MTM tissues for analyses. Because viral transduction may cause inflammation and synechiae, which could block the TM outflow pathway and lead to OHT, we first studied the morphology of the anterior segment using hematoxylin and eosin staining (Supplementary Fig. S3). We did not find obvious signs of inflammation or synechiae, and the anterior chamber angle remained open (Supplementary Fig. S3).

**Figure 6:**
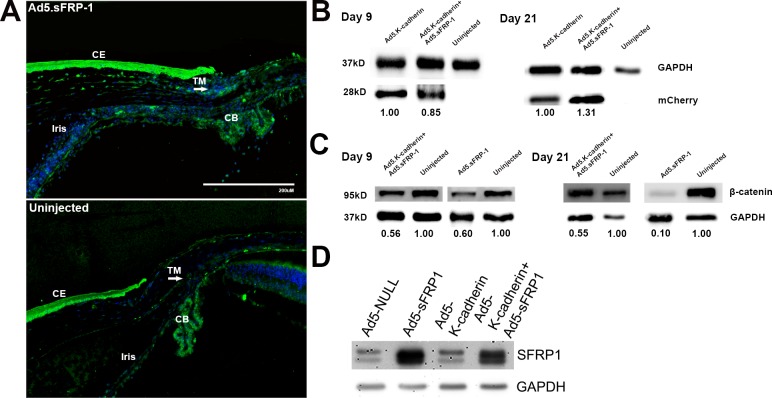
Morphological and biochemical analyses of the mouse eyes. (A) A total of 21 days postinjection, mouse eyes transduced with or without Ad5.sFRP1 were sectioned and immunostained for sFRP1 (green). Nuclei were stained with DAPI (blue). (B) and (C) On days 9 and 21 postinjection, mouse anterior segment tissues from eyes transduced with or without Ad5.sFRP1, Ad5.K-cadherin, or both were dissected, and whole-tissue lysates were extracted for WB. (D) Primary MTM cells were transduced with indicated viral particles at a multiplicity of infection of 1:100. Three days after transduction, whole-cell lysates were collected for WB.

After confirming that there was no viral-induced inflammation or chamber angle closure, we then studied the expression of sFRP1 and K-cadherin using immunofluorescence (IF) and WB, respectively. We compared sFRP1 expression using IF because it was difficult to collect aqueous humor from mouse eyes. K-cadherin was coexpressed with the mCherry protein tag and was detected by WB using anterior segment tissue lysates. Using IF staining, we found that endogenous sFRP1 was expressed in multiple tissues including the cornea, sclera, iris, ciliary body, and retina (Fig. 6A). However, there was more sFRP1 in the TM in Ad5.sFRP1 transduced eyes compared to contralateral uninjected eyes (Fig. 6A, arrow). Similarly, K-cadherin was expressed in Ad5.K-cadherin or Ad5.sFRP1+Ad5.K-cadherin transduced anterior segment tissues (Fig. 6B).

Having confirmed that sFRP1 and K-cadherin were expressed in the transduced mouse eyes, we then determined whether overexpressed sFRP1 inhibited Wnt/β-catenin signaling. We dissected anterior segment tissues and determined β-catenin levels using WB (Fig. 6C). We found that total β-catenin was decreased in the anterior segments transduced with Ad5.sFRP1 or Ad5.sFRP1 + Ad5.K-cadherin compared to contralateral uninjected control eyes.

It is possible that the inhibition of the Wnt//β-catenin signaling by K-cadherin was due to decreased sFRP1 expression resulting from cotransduction of K-cadherin. To test that possibility, we transduced cultured BALBc/J MTM cells with Ad5.Null, Ad5.sFRP1, Ad5.K-cadherin, or Ad5.sFRP1+Ad5.K-cadherin viruses.^39^ The differences in infectious viral particles were compensated using Ad5.Null virus, and multiplicity of infection was 1:100 (cell:viral colony forming units). We found that sFRP1 expression was only slightly affected by cotransduction of Ad5-K-cadherin (Fig. 6D). Therefore, it appears that the K-cadherin inhibition of sFRP1-induced OHT was not due to decreased sFRP1 expression.

## Discussion

We found that K-cadherin is highly expressed in NTM tissues and cells at mRNA and protein levels, and its expression and translocation to TM cell membrane are regulated by Wnt/β-catenin signaling. In addition, during Wnt signaling activation, some membrane associated β-catenin was localized at filopodia-like connections between TM cells, which may contribute to K-cadherin cell-cell connections. We also showed that K-cadherin contributes to TM cell-cell adhesion. Altered K-cadherin expression through induction by Wnt or inhibition by sFRP1 or siRNA significantly altered cell impedance (assessed as cell index), further supporting the roles of Wnt signaling in maintaining K-cadherin junctional function. K-cadherin overexpression significantly reduced the ocular hypertensive effects of sFRP1 in vivo. Our findings suggest that sFRP1 may induce OHT by disrupting K-cadherin, which effectively disrupts TM cell-cell adhesion and tissue homeostasis.

K-cadherin, or fetal kidney cadherin, was initially named for its high expression in the developing kidney.^40^ K-cadherin is expressed in the kidney throughout adulthood, but is also expressed in other tissues such as the ventricles of the brain and central nervous system during development and adulthood.^41–43^ Cadherins play many roles in the cell as homophilic cell-cell adhesion molecules, as cell migration promoters or inhibitors, and as mechanosensors and mechanotransducers relaying external mechanical stimulation to the actin cytoskeleton. Different cadherin isoforms may confer different physiological properties and characteristics to cells and tissues due to protein structural differences (e.g., K-cadherin has only 35% protein homology to E-cadherin^44,45^). It is necessary to further determine how K-cadherin affects TM cell behavior to understand how it regulates IOP at the molecular level. In addition to the K-cadherin expression and localization, we found that K-cadherin knockdown decreased cell impedence (CI) more than knockdown of OB-cadherin, another cadherin expressed in the TM. Interestingly, the knockdown of OB- or K-cadherin increased the expression of the other, suggesting that these two cadherins may also complement each other's function in TM cells. We need to better understand the biomechanical properties of these two highly expressed cadherins in TM cells and determine the potential functional differences between them.

**Figure 7:**
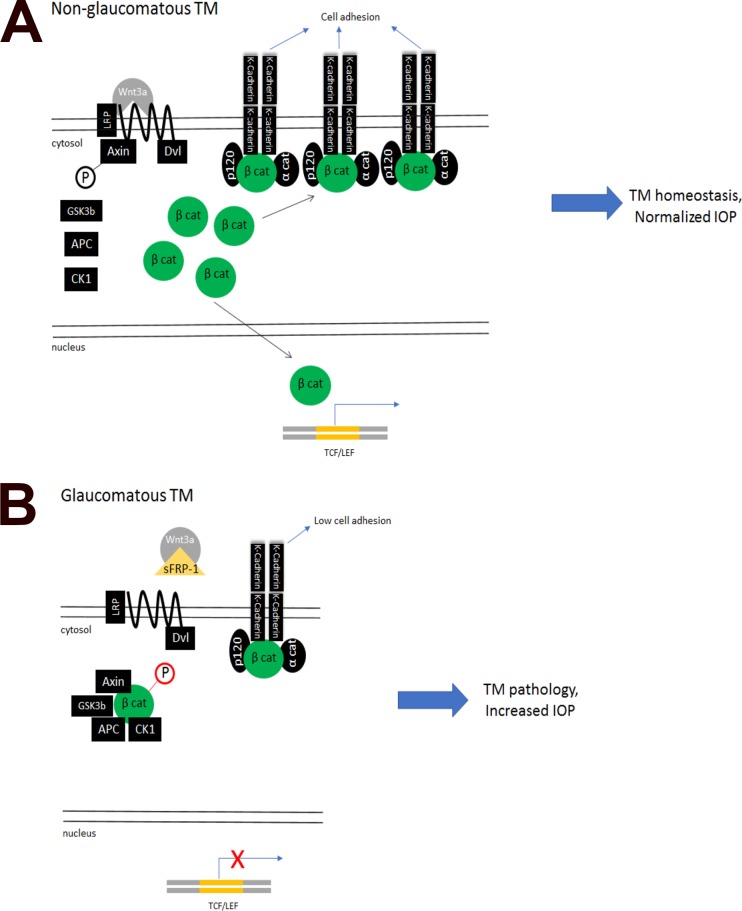
The proposed mechanism via which Wnt/β-catenin signaling regulates cell adhesion, gene expression, and IOP.

We show that active Wnt/β-catenin signaling and K-cadherin expression are important in IOP regulation, and blocking this pathway leads to IOP elevation in glaucoma (Fig. 7). Recent studies including ours show that active Wnt/β-catenin signaling inhibits fibrosis-associated proteins in the TM and that the POAG-associated Wnt antagonist sFRP1 increases ECM deposition, TM cell stiffness,^46^ and IOP.^10,19^ We also have shown that the Wnt/β-catenin signaling pathway and the TGFβ/Smad signaling pathway, another important pro-POAG pathway, cross-inhibit each other in the TM,^22^ suggesting another way by which Wnt/β-catenin signaling regulates TM homeostasis and IOP.

Future studies will determine how this cross-inhibition affects cadherin expression, TM cell adhesion, and IOP regulation. Wecker et al.^31^ examined TGFβ signaling and its effects on β-catenin as well as OB-cadherin and N-cadherin in TM cells. They found that TGFβ2 increases the expression of β-catenin, N-, and OB-cadherin in HTM cells as well as enhances TM cell-substrate adhesion.^31^ It is interesting that K-cadherin and other cadherins may work synergistically or antagonize each other during mesenchymal to epithelial transition and/or neuronal development.^41,47–49^ Based on our findings and Wecker's studies, we speculate that TGFβ signaling and Wnt signaling use OB-cadherin and K-cadherin to differentially regulate TM cell adhesion and function because they have opposing effects on IOP (i.e., increased TGFβ2 elevates IOP, whereas Wnt maintains IOP homeostasis).

Cadherin-mediated cell-cell adhesion may contribute to TM homeostasis in multiple ways. Cadherin junctions may keep cell processes in close proximity to enable gap junction formation and paracrine cell signaling. Cadherin junctions may be part of mechanical signal transduction such as contraction/relaxation to the actin cytoskeleton and may even regulate focal adhesion.^50^ Changes in the actin cytoskeleton including increased TM contractility and formation of cross-linked actin networks as well as alterations in the focal adhesion integrin molecules may contribute to increased IOP in POAG.^6,51–53^ Because we found that the Wnt/β-catenin signaling inhibitor sFRP1 decreased K-cadherin expression, it may affect K-cadherin mediated cell-cell adhesion, actin cytoskeleton, and focal adhesions and thereby regulate aqueous humor resistance via the TM outflow pathway.

## Supplementary Material

Supplement 1
