# Supplementary material for: The Role of Wnt/β-Catenin Signaling and K-Cadherin in the Regulation of Intraocular Pressure
Source: Invest Ophthalmol Vis Sci. 2018 Mar;59(3):1454–66. doi: 10.1167/iovs.17-21964 (PMC5858463; doi:10.1167/iovs.17-21964)
Supplement: Supplement 1 [file iovs-59-02-57_s01.pdf]

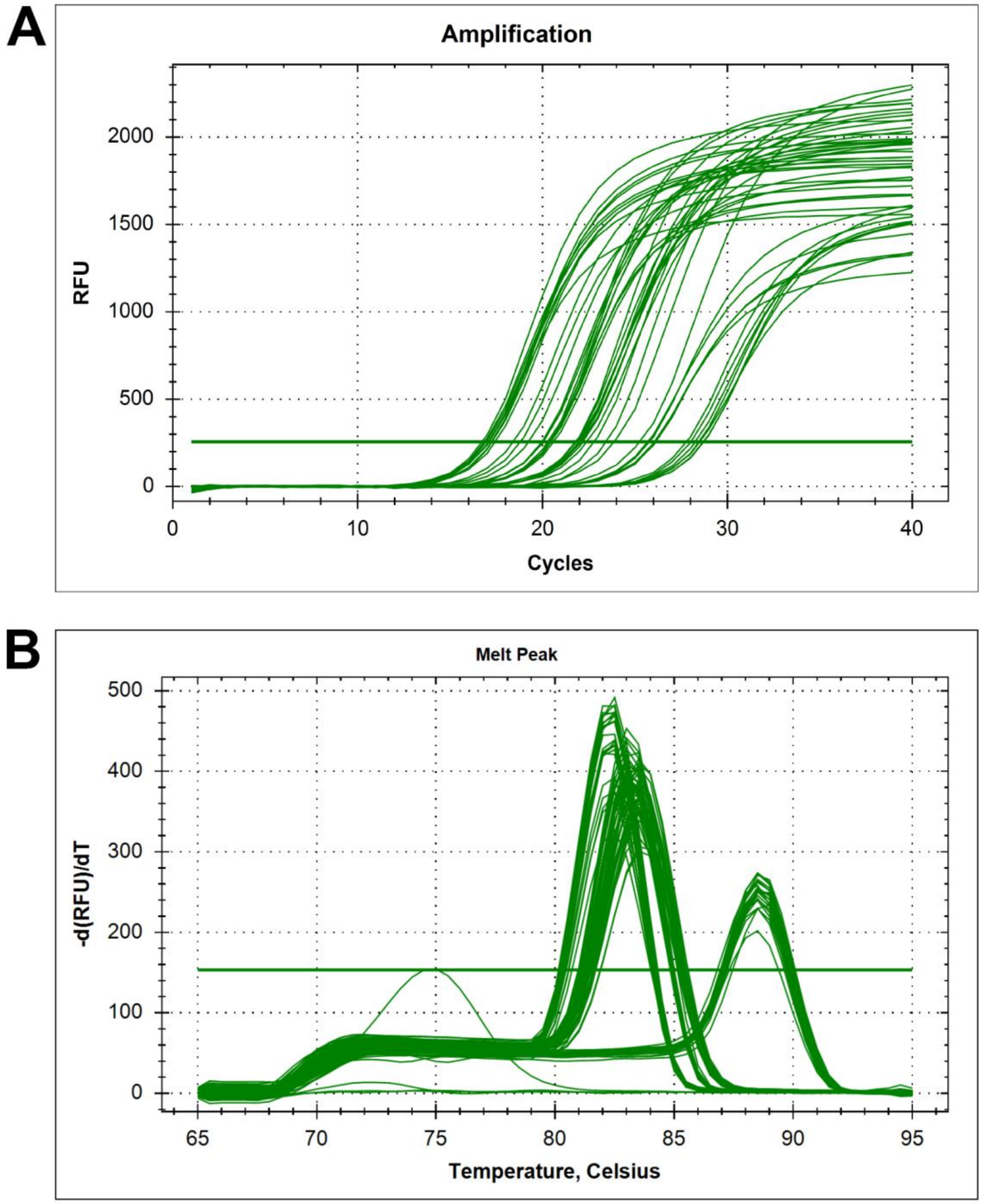

Supplemental Figure 1. Raw Amplification profiles (A) and melting curves (B) from a qPCR experiment studying the levels of cadherins, Axin2, and GAPDH in NTM cells.

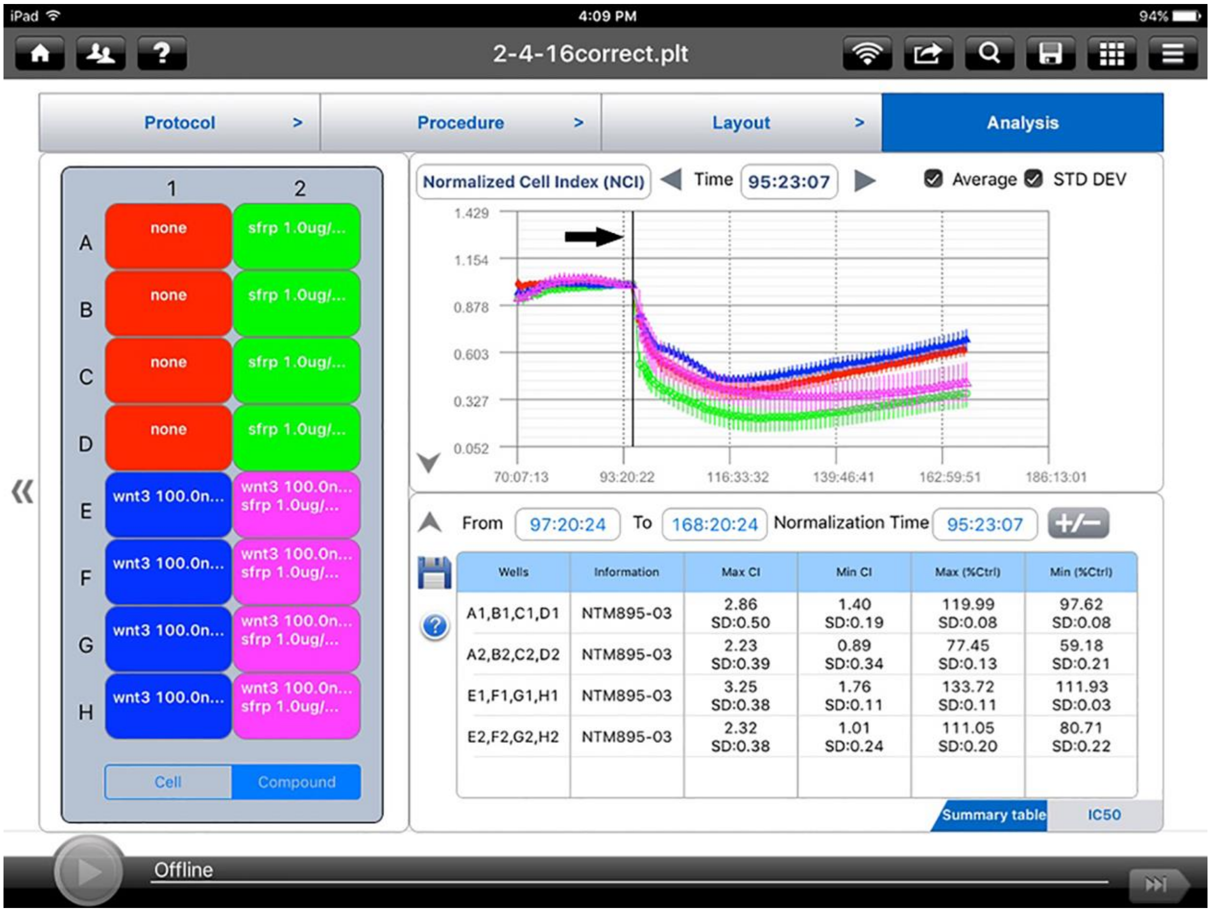

Supplemental Figure 2. Acea RTCA Normalized Cell Index values of cultured NTM cells over 72 hours. Cells were treated with or without Wnt3a and/or sFRP1 recombinant proteins.

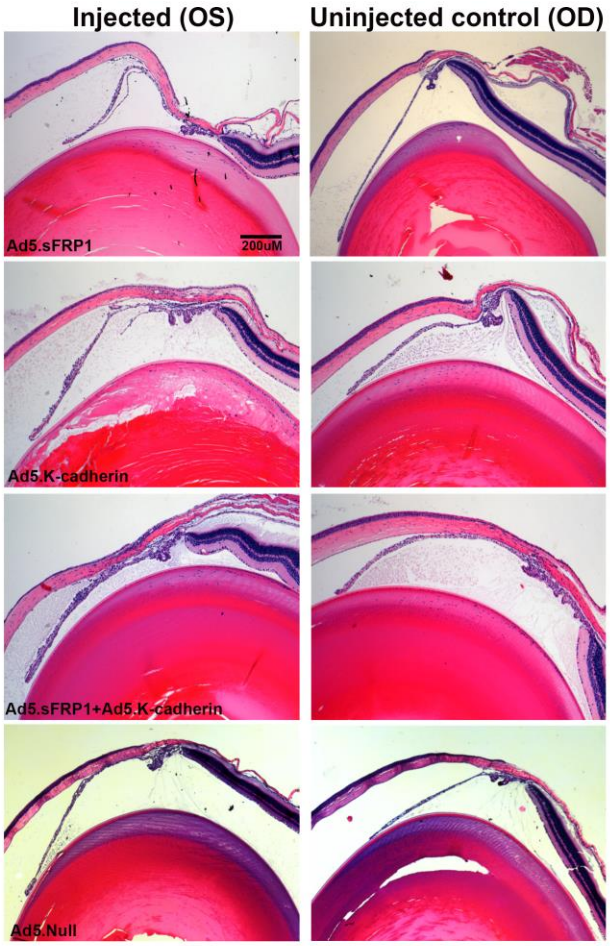

Supplemental Figure 3. H&E staining of some mouse eyes transduced with indicated Ad5 viruses. On day 35 post-injection, paired injected (OS) and uninjected contralateral control (OD) eyes from each group were enucleated, fixed, sectioned, and stained with H&E.
